# Influenza Vaccination Among Patients Undergoing Treatment for Rheumatological Disorders: Awareness, Vaccination Rates, and Influencing Factors

**DOI:** 10.5152/ArchRheumatol.2025.11077

**Published:** 2025-06-23

**Authors:** Merve Sari̇ Akyuz, Bülent Akyuz, Olgun Keski̇n, Ilhan Sezer

**Affiliations:** 1 Department of Pulmonary Medicine, Antalya Training And Research Hospital, Antalya, Türkiye; 2 Department of Rheumatology, Akdeniz University Hospital, Antalya, Türkiye

**Keywords:** Hesitancy, immunosuppressive therapy, influenza, rheumatological disorders, vaccination

## Abstract

**Background/Aims:**

Patients with autoimmune inflammatory rheumatic diseases (AIIRDs) are particularly vulnerable to infections as a result of their underlying autoimmune conditions. This vulnerability is further exacerbated by immunosuppressive treatments and associated comorbidities. This study aims to evaluate influenza vaccination rates, hesitancy, and awareness among this patient population.

**Materials and Methods:**

This descriptive study included patients with AIIRD receiving treatment at rheumatology and pulmonary medicine outpatient clinics. Between January and April 2024, a questionnaire was administered to assess influenza vaccination rates, knowledge, and attitudes.

**Results:**

Of the patients, 34.3% had received at least 1 influenza vaccination, while only 13% were vaccinated annually. Additionally, 62.2% recognized that they were at risk for influenza infection due to their current illnesses and medications and believed that they should be vaccinated. However, 59.2% had not received any professional information about the influenza vaccine. Only 38.2% were aware that vaccination was available free of charge for their condition. Older age, prolonged medication use, extended duration since diagnosis, presence of comorbidities, awareness of influenza risk, and receiving information about vaccination were all significantly associated with having received at least 1 influenza vaccination. No statistical relationship was observed between the type of rheumatic disease and vaccination (*P* = .7803). Patients relying on social media, TV, or internet sources demonstrated greater vaccine hesitancy (*P* < .0001). Awareness of vaccination recommendations was significantly associated with medication type (*P* < .0001). Hesitancy was reported by 38.7% of all patients and 48% of unvaccinated patients, influenced by negative experiences during the COVID-19 vaccination process.

**Conclusion:**

Influenza vaccination coverage among patients with AIIRD remains suboptimal. Physician reminders during routine visits could enhance vaccination rates. Health authorities might consider implementing pop-up alerts in clinical systems to prompt physicians to recommend vaccination when prescribing immunosuppressive medications.

**Main Points**

- Influenza vaccination coverage among AIIRD patients remains low: only 34.3 % received at least one dose and just 13 % are vaccinated annually.
- Older age, longer disease duration, comorbidities, and professional information all significantly increased vaccination likelihood (P < .01).
- Patients informed by healthcare providers (especially rheumatologists and primary care physicians) had markedly higher vaccination rates than those relying on social media, TV, or the internet (P < .0001).
- Negative COVID-19 vaccine experiences amplified hesitancy, affecting 48 % of unvaccinated patients (P < .0001), and younger, healthier individuals were more likely to remain unvaccinated (P  = .0016; P  = .035).
- Reminder systems—such as physician prompts during follow-ups or electronic pop-up alerts when prescribing immunosuppressives—are crucial to improve influenza vaccine uptake in this high-risk population.

## Introduction

Influenza is a respiratory viral infection that can be self-limiting but may also lead to serious complications such as pneumonia or organ failure, depending on factors like the patient’s age, comorbidities, and immunosuppressive medications.^1^ In the general population, approximately 1 in 10 unvaccinated adults are estimated to be infected with influenza each year, with half of these cases being symptomatic.^2^

Every year, approximately 1 billion cases of influenza are estimated, including 3-5 million severe cases and between 290 000 and 650 000 influenza-related respiratory deaths (with a case fatality rate ranging from 0.1% to 0.2%).^3^ Influenza vaccines potentially lead to emergency department visits and serious infections requiring hospitalization, such as pneumonia and sepsis. A meta-analysis of reports published before 2001 revealed that vaccination reduced the number of cases of influenza-like illnesses by 35%, pneumonia and hospitalizations due to influenza by 47%, and deaths from all causes by 50%.^4^

**Figure 1.**
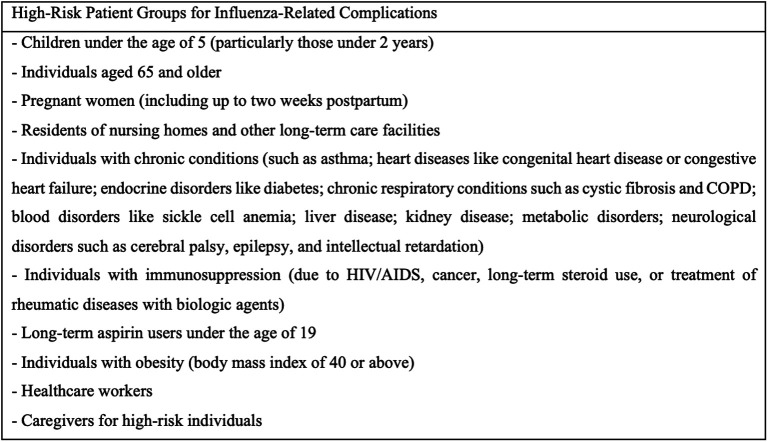
Patient groups at high risk for influenza-related complications.^5,6^

To ensure the optimal effectiveness of the vaccine against strains prevalent in both the northern and southern hemispheres, the composition of influenza vaccines is revised twice a year and adjusted according to the types of circulating influenza viruses obtained from the World Health Organization Global Influenza Surveillance and Response System. In the country, inactivated quadrivalent influenza vaccines (influenza A-B) are provided free of charge to high-risk patients for influenza-related complications, as determined by the Ministry of Health (Figure 1).

**Figure 2.**
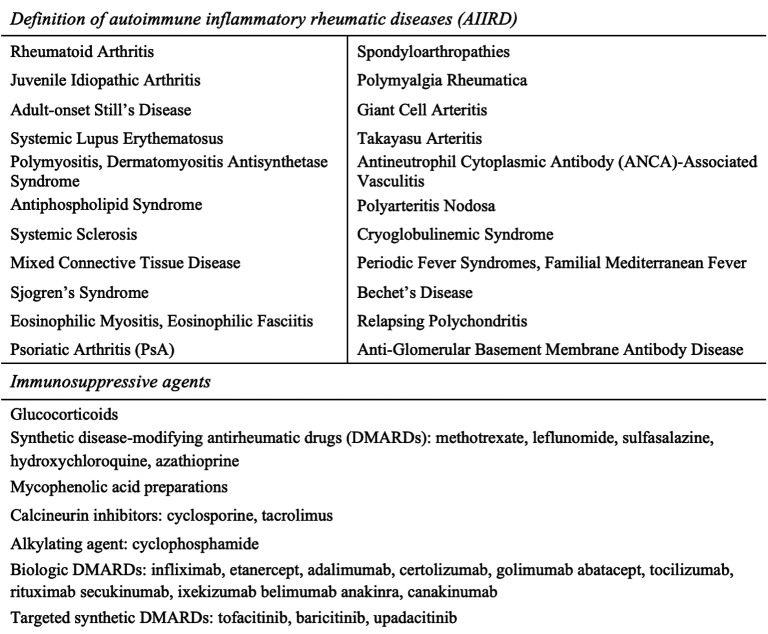
Definition of autoimmune inflammatory rheumatic diseases (AIIRDs), immunosuppressive agents (adapted from Furer et al).^7^

Patients with autoimmune inflammatory rheumatic diseases (AIIRDs) (Figure 2) are at a higher risk of influenza and its complications than the general population.^7-10^ It has been shown that influenza complications are 2.75 times more common in patients with rheumatoid arthritis (RA) than in those without RA.^9^ Additionally, infections constitute a significant cause of mortality in patients with connective tissue diseases. In a follow-up study over approximately 8 years, the overall median mortality was reported to be 20%, with mortality attributable to infections at 5.2%.^11^

Vaccination is particularly important for patients with AIIRD and can potentially lead to lower rates of hospital admissions and emergency department visits due to infections.^12^ It has been observed that vaccination is universally underutilized in the AIIRD population.^13,14^ In this patient group, the low referral rate for vaccination by rheumatologists and other treating physicians, affected by concerns about the efficacy, immunogenicity, and safety of vaccines, indicates a need to raise awareness about vaccination among healthcare professionals.^15,16^

Current guidelines recommend an annual inactivated influenza vaccine for patients with AIIRD. According to the 2019 EULAR recommendations, patients should ideally be vaccinated during periods of disease inactivity and, preferably, before the initiation of immunosuppressive treatment (Figure 2). Inactivated vaccines can be safely administered to AIIRD patients regardless of the underlying treatment. A tailored vaccination schedule should be developed for each patient.^7^ For these reasons, identifying patients’ attitudes and behaviors toward influenza vaccination, as well as assessing their level of knowledge, is crucial for informing future vaccination policies.

The primary aim of this study was to assess influenza vaccination rates, hesitancy, and awareness among patients with AIIRD. Additionally, this study sought to identify factors associated with vaccine uptake and hesitancy.

## Materials and Methods

### Study Population

This study included patients under outpatient follow-up for the AIIRD at Antalya Training and Research Hospital - Pulmonary Medicine and Akdeniz University Hospital - Rheumatology Clinic between January and April 2024. The study design was a descriptive survey. The inclusion criteria for this study encompassed patients aged 18 years and older with a confirmed diagnosis of AIIRD for at least 1 year and who were receiving appropriate immunosuppressive treatment. The exclusion criteria involved patients unwilling to participate, those with difficulty reading or understanding Turkish, and those with conditions that impaired understanding or answering the questions. All patients who met the inclusion criteria and voluntarily agreed to complete the questionnaire were included in the study.

Patients were evaluated based on their comorbidities, and the following conditions were recorded: diabetes mellitus, coronary artery disease, chronic lung disease, chronic kidney disease, chronic liver disease, and cancer.

### Questionnaire and Data Collection

A comprehensive questionnaire containing 24 items was administered to participants who provided informed consent. The questionnaire was developed by the authors based on a review of existing literature and expert opinions. This form collected demographic details (age, gender, comorbidities, occupation), specifics regarding the rheumatic disease (diagnosis, treatment, duration of disease), influenza vaccination status, level of knowledge about influenza vaccination, the source of information (doctor, social media, TV), and attitudes toward vaccinations. Data on disease and medication duration were verified through both the questionnaire and the hospital information system. Patients with discrepancies in their data were not included in the study.

Patients were primarily categorized into 2 main groups based on their vaccination status: those who had been vaccinated and those who had never been vaccinated. Among the vaccinated group, patients were further classified as those who received the influenza vaccine annually and consistently after their rheumatic disease diagnosis and those who did not receive it regularly each year.

### Ethical considerations

The study was approved by the Ethics Committee of Antalya Training and Research Hospital (Approval date: December 28, 2023, Decision no: 18/26). Participation was voluntary, and informed consent forms were obtained from all patients.

### Data Analyses and Statistical Methods

The statistical analyses of the data obtained in the study were conducted via SAS 9.4 software (SAS Institute Inc., Cary, NC, USA). Descriptive statistics are presented as the means and SDs for quantitative variables and as counts and percentages for categorical variables. The normality of the distribution of quantitative variables was assessed via the Shapiro-Wilk test and skewness coefficients. These tests revealed that the data did not follow a normal distribution; therefore, nonparametric tests were employed for statistical analysis. The Mann-Whitney *U* test was used for pairwise comparisons between 2 categorical variables, whereas the Kruskal-Wallis test was applied to identify differences among variables with 3 or more categories.

When a significant difference was detected in the Kruskal-Wallis test, the Mann-Whitney *U* test was performed for pairwise comparisons to identify the source of the difference.

Chi-square analysis was used to examine relationships among categorical variables. When a significant difference was found, the Bonferroni test was conducted to identify the source of the difference among independent categorical groups.

Stepwise regression was used to build a regression model to describe and identify the independent variables affecting the dependent variables. Stepwise regression was used to find the best predictor among all the significant predictors for this dataset, 1 for each response variable. Stepwise regression is a procedure used to build a model in successive steps. In stepwise regression, explanatory variables can be added or deleted at each step. The selection criteria are common for linear regression. An F test and a test of the significance of each variable was used on the variable added or deleted from the model at each step. Odds ratios, confidence intervals, and significance levels were derived from this analysis. Throughout the study, *P* < .05 was considered statistically significant. 

### Definitions

Marking any of the following responses “I am concerned about the side effects of the vaccine,” “I don’t think I need it,” “I am worried about harmful ingredients in the vaccine,” “I don’t believe getting vaccinated is appropriate,” and “I am concerned about a flare-up of my rheumatic disease” were defined as the presence of vaccine hesitancy.

Patients were classified into the following categories according to their medication use: anti-TNF (etanercept, adalimumab, infliximab, certolizumab pegol, and golimumab), conventional synthetic disease-modifying antirheumatic drugs (csDMARDs) (methotrexate, hydroxychloroquine, sulfasalazine, leflunomide, azathioprine, cyclosporine and tacrolimus), targeted synthetic DMARDs (tsDMARDs) (tofacitinib, baricitinib, and upadacitinib), rituximab (RTX), other biologic agents (tocilizumab, abatacept, anakinra, canakinumab, secukinumab, ustekinumab, and ixekizumab) and mycophenolate mofetil (MMF). Combination treatment refers to the concurrent use of anti-TNF agents, tsDMARDs, other biologic agents, or RTX alongside any csDMARD.

## Results

A total of 463 patients were included in the study, with an average age of 47.4 years (range: 18-83), and 252 (54.4%) of them were female. The patients had an average duration of disease of 11.4 years and an average duration of medication usage of 7.2 years. A total of 117 patients (25.3%) had comorbid conditions in addition to their rheumatic disease, with diabetes mellitus being the most common comorbidity at 12.3% (57 patients). The majority of patients were diagnosed with axial spondyloarthritis (axSpA) at 51.8% and RA at 27.4%. In terms of treatment, 65.7% of patients were receiving anti-TNF treatment, 21.2% were receiving conventional DMARDs, and a smaller percentage were receiving other medications. Additionally, 14.9% of patients were using low-dose corticosteroids, while 25.7% were receiving combination treatment (Table 1).

Among the patients, 288 (62.2%) were aware that they were at risk of influenza infection due to their existing condition and medications and recognized the need for vaccination. However, 274 (59.2%) had never received any information about the influenza vaccine. A total of 34.3% had received the influenza vaccine at least once, with only 13% being vaccinated annually. When asked about their opinions on the influenza vaccine, 38.7% were unaware that vaccination was recommended, while 21.4% felt it was unnecessary. Moreover, 38.2% of the patients believed the vaccine was beneficial, and 13.8% were concerned about the exacerbation of their rheumatic disease. In response to why they might choose to receive the influenza vaccine, 62.6% of the participants reported self-protection as their primary reason, 38.2% cited their chronic condition, and 34.8% indicated that they would do so upon a doctor’s recommendation. Of the patients who had previously been informed about the influenza vaccine, 54% received information from a rheumatologist, 45% from a primary care physician, 22.8% from a pulmonologist, and 20.1% from social media, TV, radio, or the internet. Only 38.2% knew that vaccination was provided free of charge by the state for patients with their conditions. Among the patients who had not been vaccinated, 58.6% reported that they would consider vaccination after completing this questionnaire. Additionally, 38.7% of the patients expressed hesitation toward vaccines following the COVID-19 pandemic (Table 2).

A significant association was found between having received the vaccine at least once and factors such as older age, longer duration of medication use and disease, presence of comorbidities, awareness of influenza risk, and having been informed about the vaccine. Unmarried individuals were more likely not to have been vaccinated (*P* = .0381), while no significant differences were found regarding vaccination rates in terms of gender, education level, or income. Unvaccinated patients had a higher frequency and severity of influenza infections (*P* < .0001). No significant association was found between the type of rheumatic condition and vaccination (*P* = .7803). Similarly, the type of antirheumatic medication, corticosteroid use, or combination treatment had no correlation with receiving the influenza vaccine (Table 3).

In the question regarding the source of information about vaccination, the rate of influenza vaccination was significantly higher among patients who received information from their rheumatologist, primary care physician, pulmonologist, pharmacist, other healthcare personnel, or other doctors, whereas those who obtained information through social media, TV, or the internet had a higher rate of not being vaccinated. Among the unvaccinated patients, 81.3% were unaware that vaccines were provided free of charge by the government (*P* < .0001). Additionally, 48% of unvaccinated patients reported reluctance toward influenza vaccination due to negative perceptions shaped by the COVID-19 vaccination process (*P* < .0001) (Table 3).

Vaccine hesitancy was more prevalent among younger patients and those without additional comorbidities (*P* = .0016, *P* = .035). The rate of hesitancy was lower in patients who had received information about the vaccine (*P* = .0006). There was no significant association between hesitancy and the type of disease or treatment received (*P* = .588, *P* = .114). Patients who relied on social media/TV/internet as their source of vaccine information exhibited higher rates of hesitancy (*P* < .0001). Among the patients with vaccine hesitancy, 61.1% expressed reservations toward influenza vaccines following the COVID-19 pandemic (*P* = .0001).

In response to this question, “What are your thoughts on the influenza vaccine?” which allowed for multiple selections, 53% of unvaccinated patients were unaware of the recommendation for vaccination, 29.9% felt they did not need it, 24% did not know how to obtain the vaccine, and 18% believed that vaccination was unnecessary (*P* < .0001). Common reasons for vaccination among patients included the desire to protect themselves, and their families and the need due to chronic conditions (*P* < .0001) (Table 3). Patients who cited “I am concerned about side effects” (*P* = .0419), “I do not think I need it” (*P* < .0001), or “I do not believe that getting vaccinated is appropriate” (*P* < .0001) as reasons tended to answer “no” when asked if they would consider vaccination following this questionnaire. For those who expressed vaccine hesitancy related to the COVID-19 pandemic, the majority reported no change in their perspective on influenza vaccination after the questionnaire (*P* < .0001).

In the subgroup analysis between those receiving vaccinations regularly and irregularly, patients who were vaccinated regularly experienced illness with milder symptoms (*P* = .0003). Within this patient group, there was a statistically significant association between regular vaccination and factors such as older age, longer duration of disease, and the presence of comorbidities (*P* < .0001, *P* = .251, *P* = .0005). Patients informed about the vaccine by their primary care physician had a significantly higher rate of regular annual vaccination, whereas no such association was found with other sources of information (*P* = .01). Among patients vaccinated irregularly, 29.3% stated that their hesitancy toward influenza vaccination was affected by negative experiences with the COVID-19 vaccination process (*P* = .0006).

In the analysis of patients who had never received the influenza vaccine based on the medications they used, those receiving anti-TNF-α and csDMARD treatment had more severe disease (*P* < .0001, *P* = .0061) (Table 4).

In the assessment of patients’ awareness regarding their influenza infection risk due to the AIIRD and the medications they used, those who were older, had a longer disease duration, or had comorbid diseases were significantly more likely to be informed (*P* = .0187, *P* = .0074, *P* = .0008). Patients diagnosed with axSpA, RA, and psoriatic arthritis (PsA) were frequently aware of their infection risk and the recommendation for vaccination, whereas a substantial portion of patients with Sjogren’s syndrome were unaware of this recommendation (*P* = .0009). Among those who knew that they were at risk and that vaccination was recommended, only 46.5% had been vaccinated at least once, and merely 18.1% were vaccinated annually (*P* < .0001). Additionally, 75.7% of this informed patient group answered “yes” to the following question: “Would you consider getting vaccinated after this questionnaire?” (*P* = .0001). Among patients without this information, 82.3% were also unaware that the vaccination was provided by the government (*P* < .0001). Patients receiving anti-TNF and RTX treatments were more likely to be informed about the vaccination recommendation, whereas those receiving csDMARDs were less likely to know of this recommendation (*P* < .0001).

A significant association was identified between responding “yes” to the question, “Have you been informed about the influenza vaccine?” and various factors, including older age (*P* < .0001), longer duration of medication use (*P* = .0026), extended duration since diagnosis (*P* = .0004), and the presence of comorbidities (*P* = .0019). Among individuals who had received information about the vaccine, 68.3% reported being vaccinated at least once, and 28% indicated adherence to annual vaccination schedules (*P* < .0001). Furthermore, 72% of informed respondents were aware that the vaccine was provided free of charge by the government (*P* < .0001), and 70.4% expressed positive attitudes toward vaccination, influenced by the COVID-19 pandemic (*P* = .0009). Notably, among those who had not been informed about influenza vaccination, 63.1% stated that they would consider receiving the vaccine following their participation in the questionnaire (*P* = .0005).

A significant relationship was also identified between the severity of influenza infection and factors such as older age, duration of medication use, and duration of disease (*P* = .014, *P* = .0096, *P* < .0001). Among patients with mild influenza, 10.8% were regular corticosteroid users, whereas 26.9% of those with severe influenza were regular corticosteroid users (*P* < .0001). Patients who had not received the influenza vaccine experienced more severe symptoms (*P* < .0001). The severity of influenza was higher among those using MMF, RTX, or combination treatment compared to those using other medications (*P* = .0013, *P* = .0005).

Patients receiving combination treatment were older on average; 82.4% were female, and 35.3% had comorbidities. Additionally, 66.4% of the patients were diagnosed with RA. A significant association was observed between receiving combination treatment and both the severity and frequency of influenza infection (*P* = .0005, *P* = .0013).

The logistic regression analysis evaluated 3 distinct questions (Table 5). For the question “Do you receive the influenza vaccine?” with binary responses (yes/no), multiple factors were significantly associated with vaccination status. Age was a positive predictor, with increasing age leading to higher vaccination rates (OR: 1.048, 95% CI: 1.018-1.079, *P* = .0015). Being informed about influenza vaccination substantially increased the likelihood of receiving the vaccine (OR: 6.647, 95% CI: 3.286-13.448, *P* < .0001). The perception that the vaccine reduces the risk of severe illness was also a significant factor (OR: 6.522, 95% CI: 2.737-15.542, *P* < .0001). Conversely, unawareness of the vaccine recommendation (OR: 0.167, 95% CI: 0.078-0.358, *P* < .0001) or considering vaccination unnecessary (OR: 0.200, 95% CI: 0.069-0.579, *P* = .0030) were associated with lower vaccination rates. Additionally, concerns about influenza vaccination arising after the COVID-19 pandemic were significant, with these concerns increasing the likelihood of vaccination (OR: 4.498, 95% CI: 1.881-10.755, *P* = .0007). For the question “Influenza infection severity? “ the responses ranged from no work disruption to requiring hospital care. Significant predictors included corticosteroid use (OR: 4.146, 95% CI: 2.017-8.522, *P* = .0001) and duration of disease (OR: 1.093, 95% CI: 1.055-1.133, *P* < .0001), indicating that patients with longer disease histories or corticosteroid treatments were more likely to report severe outcomes. In the question “Are you aware that you are at risk due to my rheumatic disease and that treatment and vaccination is recommended?” significant predictors were comorbidity (OR: 2.269, 95% CI: 1.250-4.120, *P* = .0071) and duration of medication use (OR: 1.093, 95% CI: 1.021-1.170, *P* = .0101).

## Discussion

Our study revealed that while 34.3% of the patients had received an influenza vaccination at least once, only 13% were vaccinated regularly each year. The vaccination rate was 36.2% for RA patients and 33.75% for axSpA patients. In the literature, influenza vaccination rates for RA patients alone range between 25% and 90%.^17^ In a study conducted in Germany with a cohort including AIIRD patients, the overall influenza vaccination rate was reported to be 68.5%, with the majority of patients being diagnosed with RA, among whom 71.1% were vaccinated.^18^ The variations in vaccination rates across countries reflect differences in vaccination programs, cultural factors, and study designs.

In the literature, RA patients generally have higher vaccination rates, which could be attributed to factors such as older age, combined treatment, and steroid use in this group. Notably, in this study, 66.4% of RA patients received combination treatment. However, there was no significant difference in vaccination rates between RA patients and other patients in this study. The findings indicate that influenza vaccine coverage remains low among AIIRD patients, particularly among older RA patients and those receiving combination treatment, underscoring the need to expand vaccination policies as an essential aspect of their care.

Studies have shown that the SARS-CoV-2 vaccination rate among AIIRD patients is high and that the COVID-19 pandemic has positively impacted the uptake of other vaccinations as well.^19^ In a cross-sectional study conducted in Greece, the influenza vaccination rate among AIIRD patients was reported to be 83%, with this high rate associated with the positive influence of the COVID-19 pandemic on attitudes toward vaccination.^20^ However, hesitancy toward the COVID-19 vaccine may arise from more complex and varied reasons than those associated with influenza vaccination. Notably, the large-scale impact of the COVID-19 pandemic and widespread misinformation have contributed to an increase in negative perceptions about vaccination.^21^ In this study, 38.7% of the general population and 48% of those unvaccinated against influenza indicated that they had become more hesitant about vaccines after the COVID-19 pandemic, which may be another factor in the lower vaccination rate within the population. Among those with post-pandemic reservations about vaccinations, a significant proportion showed no change in their perspective on influenza vaccination after the questionnaire, suggesting that vaccine resistance persists within certain subgroups. The literature has shown that individuals with hesitancy toward both COVID-19 and influenza vaccines exhibit similar behavioral patterns within specific subgroups.^22^

Our study’s patient population is predominantly composed of individuals with axSpA, a condition more frequently observed in younger populations.^23^ As a result, it can be inferred that the average age is lower compared to the literature. To the authors’ knowledge, studies related to vaccination are often conducted primarily in RA populations, with insufficient attention given to patients diagnosed with axSpA. In the literature, a questionnaire-based cross-sectional study involving 199 patients with axial SpA reported an influenza vaccination rate of 20.1%.^24^ In contrast, the findings indicate that the rate of influenza vaccination among patients with axSpA is 33.75%, which is higher than that reported in the literature. A randomized controlled meta-analysis involving patients with SpA and AS noted a 1.57-fold higher risk of serious infections among those using anti-TNF agents than among those in the placebo group, although this difference was not statistically significant.^25^ Conversely, a systematic review and meta-analysis including RA, AS, and PsA patients reported a significant increase in the risk of infections and serious infections among those using anti-TNF agents.^26^ These findings suggest that healthcare providers should recommend vaccination for all AIIRD patients, irrespective of subtype. Since axSpA patients can only be treated with non-steroidal anti-inflammatory drugs (NSAIDs), it is believed that vaccination reminders may be overlooked during visits when immunosuppressive treatment is initiated.

Vaccination rates among outpatients can be enhanced through regular reminders and education on vaccination during follow-up and consultations.^27^ In this study, patients informed by healthcare professionals had higher influenza vaccination rates. The vaccination rate among patients who had previously been informed about the vaccine was almost double the overall patient vaccination rate. These findings suggest that physicians should more actively recommend vaccination during visits. Previous studies have indicated that the primary reason patients are not vaccinated against influenza is the lack of recommendations from their doctors.^16,28^ Recently, it has been reported that rheumatologists play a significant role in reducing vaccine hesitancy; however, primary care physicians are the main vaccination providers, emphasizing the need for collaboration.^29^ Patients who recognized that they were at risk and needed vaccination reported a 75.7% likelihood of considering vaccination after the questionnaire. This result indicates that reminders about vaccination during repeated visits can significantly increase vaccination uptake. Previous research has also shown that multimodal strategies, such as email reminders and recommendations from physicians, are effective in increasing vaccination rates.^30^

The relationship between treatment type and vaccination remains uncertain. In a study involving patients with SLE, a significant association was reported between influenza vaccination and the use of corticosteroids equivalent to ≥ 7.5 mg of prednisone.^31^ Conversely, another study reported no significant relationship between treatment type and vaccination rates.^32^ In this study, no significant relationships were detected between the type of disease, corticosteroid use, or the application of combination treatment and vaccination rates.

A limitation of this study is that the vaccination status was self-reported by the patients, and there were no medical records regarding vaccination. However, the literature suggests that self-reported vaccination information can adequately represent actual vaccination records, although it may overestimate coverage by approximately 10%.^33^

As a result of this study, it is believed that influenza vaccination coverage among patients with AIIRD is low. Encouraging patients to get vaccinated during follow-up visits and ensuring proper vaccination conditions are crucial steps to reduce transmission risk and prevent complications. The Ministry of Health could implement pop-up alerts to remind physicians to recommend vaccination to their patients when immunosuppressive medications are prescribed.

## Figures and Tables

**Table 1.** Demographic Characteristics

	Total (n = 463)
Age	
Mean (SD), median (range)	47.4 (13.00), 47.0 (18.0, 83.0)
Duration of medication (years)	
Mean (SD), median (range)	7.2 (5.26), 6.0 (1.0, 41.0)
Disease duration (years)	
Mean (SD), median (range)	11.4 (8.06), 10.0 (1.0, 45.0)
Gender, n (%)	
Female	252 (54.4)
Education, n (%)	
Primary-secondary education	199 (43.0)
High school	153 (33.0)
> High school	111 (24.0)
Marital status, n (%)	
Married	365 (78.8)
Income status, n (%)	
Income less than expenses	168 (36.3)
Income equal to expenses	246 (53.1)
Income more than expenses	49 (10.6)
Comorbidity, n (%)	117 (25.3)
Diabetes mellitus, n (%)	57 (12.3)
Coronary artery disease, n (%)	30 (6.5)
Chronic lung disease, n (%)	31 (6.7)
Chronic kidney disease, n (%)	9 (1.9)
Chronic liver disease, n (%)	2 (0.4)
Cancer history, n (%)	3 (0.6)
Diagnosis, n (%)	
Axial spondyloarthritis	240 (51.8)
Rheumatoid arthritis	127 (27.4)
Psoriatic arthritis	28 (6.0)
Systemic lupus erythematosus	26 (5.6)
Systemic sclerosis	5 (1.1)
Sjogren’s disease	10 (2.2)
Familial mediterranean fever	22 (4.8)
ANCA associated vasculitides	1 (0.2)
Behçet’s Disease	2 (0.4)
Dermatomyositis	2 (0.4)
Medication, n (%)	
Anti-TNF	304 (65.7)
csDMARDs	98 (21.2)
Other biologics drugs	23 (5.0)
Targeted synthetic DMARDs	12 (2.6)
Mycophenolate mofetil	13 (2.8)
Rituximab	13 (2.8)
Corticosteroid use, n (%)	69 (14.9)
Combination treatment, n (%)	119 (25.7)

csDMARDs, conventional synthetic disease-modifying antirheumatic drugs; DMARDs, disease-modifying antirheumatic drugs.

**Table 2.** Questionnaires

I am aware that I am at risk due to my rheumatic disease and treatments and vaccination is recommended, n (%)	288 (62.2)
I have been informed about the influenza vaccine., n (%)	189 (40.8)
I get the influenza vaccine, n (%)	159 (34.3)
Severity of influenza infection, n (%)	
I did not experience any loss of work	344 (74.3)
I was unable to continue working and had to visit the hospital for medical care	119 (25.7)
What do you think about the influenza vaccine? n (%)	
It is beneficial	177 (38.2)
Reduces the risk of serious illness	91 (19.7)
I was not aware it was recommended	179 (38.7)
I am concerned about side effects of the vaccine	68 (14.7)
I do not think I need it	99 (21.4)
I am worried about harmful ingredients in the vaccine	40 (8.6)
I do not know how to obtain the vaccine	85 (18.4)
I don’t believe getting vaccinated is appropriate	60 (13.0)
I am concerned about a flare-up of my rheumatic disease	64 (13.8)
Where did you receive information about the vaccine? n (%)	
Primary care physician	85 (45)
Family and social circle	26 (13.8)
Pharmacist	28 (14.8)
Other healthcare personnel	22 (11.6)
Pulmonologist	43 (22.8)
Rheumatologist	102 (54)
Other doctors	43 (22.8)
Social media/TV/Internet	38 (20.1)
Are you aware that the vaccine is covered by the government for those with rheumatic diseases and those undergoing treatment? n (%)	
Yes	177 (38.2)
After this questionnaire, if you have not been vaccinated, would you consider getting vaccinated? n (%)	
Yes	178 (58.6)
After the COVID-19 pandemic, do you have any concerns about getting the influenza vaccine? n (%)	
Yes	179 (38.7)

**Table 3.** Comparison of Demographic and Attitudinal Characteristics of Patients By Vaccination Status

	Did You Receive the Influenza Vaccine?		*P*
	Yes (N = 159)	No (N = 304)	
Age, mean (SD)	52.6 (12.58)	44.7 (12.41)	<.0001^1^
Duration of medication (years), mean (SD)	7.9 (5.44)	6.8 (5.14)	0.0081^1^
Disease duration (years), mean (SD)	12.9 (8.58)	10.6 (7.67)	0.0034^1^
Gender, n (%)			0.1426^2^
Female	94 (59.1)	158 (52.0)	
Education, n (%)			0.3678^2^
Primary-secondary education	71 (44.7)	128 (42.1)	
High school	46 (28.9)	107 (35.2)	
> High school	42 (26.4)	69 (22.7)	
Marital status, n (%)			0.0381^2^
Married	134 (84.3)	231 (76.0)	
Single	25 (15.7)	73 (24.0)	
Income status, n (%)			0.4046^2^
Comorbidity, n (%)	60 (37.7)	57 (18.8)	<.0001^2^
Diagnosis, n (%)			0.7803^2^
Corticosteroid use, n (%)	24 (15.1)	45 (14.8)	0.9333^2^
I am aware that the vaccine is covered by the government for those with rheumatic diseases and receiving treatment., n (%)	134 (84.3)	154 (50.7)	<.0001^2^
Informed about influenza vaccination, n (%)	129 (81.1)	60 (19.7)	<.0001^2^
Severity of influenza infection? n (%)			<.0001^2^
No work loss	142 (89.3)	202 (66.4)	
Unable to work, had to seek hospital care	17 (10.7)	102 (33.6)	
Thoughts on influenza vaccine			
Beneficial, n (%)	122 (76.7)	55 (18.1)	<.0001^2^
Reduces severe illness risk, n (%)	72 (45.3)	19 (6.3)	<.0001^2^
Wasn’t aware it was recommended, n (%)	18 (11.3)	161 (53.0)	<.0001^2^
Concerned about side effects, n (%)	19 (11.9)	49 (16.1)	0.2289^2^
Don’t think it’s necessary, n (%)	8 (5.0)	91 (29.9)	<.0001^2^
Concerned about harmful ingredients, n (%)	12 (7.5)	28 (9.2)	0.5452^2^
Don’t know how to obtain vaccine, n (%)	11 (6.9)	74 (24.3)	<.0001^2^
Don’t believe vaccination is appropriate, n (%)	4 (2.5)	56 (18.4)	<.0001^2^
Concerned about flare-ups of rheumatic disease, n (%)	20 (12.6)	44 (14.5)	0.5748^2^
Sources of information about vaccination			
Primary care physician, n (%)	76 (47.8)	56 (18.4)	<.0001^2^
Family and social circle, n (%)	18 (11.3)	47 (15.5)	0.2234^2^
Pharmacist, n (%)	24 (15.1)	20 (6.6)	0.0030^2^
Other healthcare personnel, n (%)	23 (14.5)	21 (6.9)	0.0085^2^
Pulmonologist, n (%)	36 (22.6)	46 (15.1)	0.0444^2^
Rheumatologist, n (%)	88 (55.3)	134 (44.1)	0.0212^2^
Other doctors, n (%)	38 (23.9)	33 (10.9)	0.0002^2^
Social media/TV/Internet, n (%)	21 (13.2)	117 (38.5)	<.0001^2^
Aware vaccine is covered by government? n (%)	120 (75.5)	57 (18.8)	<.0001^2^
Would you consider getting vaccinated after this questionnaire? n (%)	143 (89.9)	178 (58.6)	<.0001^2^
Do you have concerns about influenza vaccination after COVID-19 pandemic? n (%)	33 (20.8)	146 (48.0)	<.0001^2^
Medication, n (%)			0.0718^2^
Combination treatment, n (%)	45 (28.3)	74 (24.3)	0.3545^2^

^1^Kruskal–Wallis *P* value.

^2^Chi-square *P* value.

**Table 4.** Comparison of Influenza Infection Severity Among Patients with Medication Types and Vaccination Status

	Medications	Did You Receive the Influenza Vaccine?		*P*
		Yes	No	
Influenza infection severity? I was unable to continue working and had to visit the hospital for medical care, n (%)	Antı-TNF	(n = 106), 7 (6.6%)	(n = 198), 58 (29.3%)	<.0001^1^
	csDMARDs	(n = 25), 2 (8.0%)	(n = 73), 27 (37.0%)	0.0061^1^
	Other Biologics Drugs	(n = 9), 1 (11.1%)	(n = 14), 5 (35.7%)	0.1897^1^
	Targeted Synthetic DMARDs	(n = 4), 0 (0.0%)	(n = 8), 4 (50.0%)	0.0833^1^
	MMF	(n = 7), 3 (42.9%)	(n = 6), 3 (50.0%)	0.7968^1^
	RTX	(n = 8), 4 (50.0%)	(n = 5), 5 (100.0%)	0.0574^1^

Antı-TNF, anti-tumor necrosis factor; csDMARDs: conventional synthetic disease-modifying antirheumatic drugs; MMF, mycophenolate mofetil; RTX, rituximab.

^1^Chi-square *P* value.

**Table 5.** Logistic Regression Analysis of Factors Influencing Influenza Vaccination Status

	StdEr	Wald ChiSq	*P*	Odds Ratio	Lower CL	Upper CL
Do you receive the influenza vaccine?						
Intercept	0.8264	24.5845	<.0001			
Age	0.0148	10.1021	0.0015	1.048	1.018	1.079
Informed about influenza vaccination	0.3595	27.7607	<.0001	6.647	3.286	13.448
Thoughts on influenza vaccine						
Reduces severe illness risk	0.4430	17.9154	<.0001	6.522	2.737	15.542
Wasn’t aware it was recommended	0.3883	21.2038	<.0001	0.167	0.078	0.358
Don’t think it’s necessary	0.5436	8.7856	0.0030	0.200	0.069	0.579
Aware vaccine is covered by government?	0.3656	4.6904	0.0303	2.207	1.078	4.519
Do you have concerns about influenza vaccination after COVID-19 pandemic?	0.4448	11.4293	0.0007	4.498	1.881	10.755
Influenza infection severity?						
Intercept	0.4092	30.3980	<.0001			
Disease duration (years)	0.0182	23.9533	<.0001	1.093	1.055	1.133
Corticosteroid use	0.3676	14.9667	0.0001	4.146	2.017	8.522
Are you aware that you are at risk due to my rheumatic disease and treatments and vaccination is recommended?						
Intercept	1.2600	2.5958	0.1071			
Duration of medication (years)	0.0347	6.6101	0.0101	1.093	1.021	1.170
Disease duration (years)	0.0208	3.0923	0.0787	0.964	0.925	1.004
Comorbidity	0.3044	7.2488	0.0071	2.269	1.250	4.120
I have been informed about the influenza vaccine	0.3042	67.9362	<.0001	12.268	6.759	22.268

## Data Availability

The datasets used and/or analyzed during the current study are available from the corresponding author on reasonable request.
